# 

**Published:** 1880-08-20

**Authors:** 


### TABLE OF CONTENTS.

ORIGINAL AND SELECTED ARTICLES.

Snake Bites, by Joseph Jones, M.D., of Georgia, 273. Antiseptic Treatment of Gunshot Wound of the Knee-Joint, by J. A. Holloway, M.D., of La., 274. Artificial Abortion, by M. H. Jordan, M.D., 275. Hypodermic Medication, by T. J. Tyner, M.D., Memphis, Tenn., 278. Veratrum Viride, by W. C. Norwood, M.D., S. C., 283. Recent Progress in Obstetrics, by W. I. Richardson, M.D., 285. Operation for the Cure of Hydrocele, by Bernard Bartow, M.D., 287. Report of Clinic, from St. John's Hospital by J. Emory Lamphear, 289.

ABSTRACTS AND GLEANINGS.

Gelsemium, 291. Glycerin in Flatulence, Acidity, etc., 292. Rapid Breathing as a Pain-Obtunder in Minor Surgery, Obstetrics, etc., 294. Against the Fever; Chloride of Calcium for Phthisis, 296. Iodide of Ethyl in Dyspnœa, 297. Toxical Effects of Tea, 298. Eucalyptus Globulus; On Tonga, 299. Diabetes and Sepsis, 300. Wolfe's Operation for Cataract; Ovariotomy, 301. What becomes of Blood, etc., in the Joints; Asthma; A Certain Remedy for Diphtheria, 302.

INDEX MEDICUS

SCIENTIFIC ITEMS.

Explosive Combinations in Pharmacy, 303. Anditory Nerve; The Polyscope; Bones and Sugar, 304.

PRACTICAL NOTES AND FORMULÆ.

Vomiting of Pregnancy; Hay Fever; For External Piles, 305. Alcohol and Creosote in Infants' Diarrhœa; To Relieve Itching; Tonic Glycerine; Application for Painful Hemorrhoids; Fetid Perspiration of the Feet, 306. Cholera and Diarrhœa; For Amenorrhœa; Enema for Dysentery; Chronic Constipation; Remedies for Purpura, 307. Ice in Typhoid Fever; Cough Mixture; Chloroform Cough Mixture; Poisoning by Rhus Toxicodendron; Eucalyptus for Wounds; Picrotoxin in Night Sweats; Inquiry about Pessaries, 308.

EDITORIAL AND MISCELLANEOUS.

Editorial Notices, 309. Doctor Tanner's Fast, 310. Book Notices, 311. Receipted; Special Notices, 312.

